# Comparison of Longitudinal Changes of Cerebral Small Vessel Disease Markers and Cognitive Function Between Subcortical Vascular Mild Cognitive Impairment With and Without *NOTCH3* Variant: A 5-Year Follow-Up Study

**DOI:** 10.3389/fneur.2021.586366

**Published:** 2021-02-25

**Authors:** Cindy W. Yoon, Young-Eun Kim, Hee Jin Kim, Chang-Seok Ki, Hyejoo Lee, Joung-Ho Rha, Duk L. Na, Sang Won Seo

**Affiliations:** ^1^Department of Neurology, Inha University School of Medicine, Incheon, South Korea; ^2^Department of Laboratory Medicine, Hanyang University College of Medicine, Seoul, South Korea; ^3^Department of Neurology, Samsung Medical Center, Sungkyunkwan University School of Medicine, Seoul, South Korea; ^4^Genome Research Center, Green Cross Genome, Yong-in, South Korea

**Keywords:** *NOTCH3*, CADASIL, lacune, cerebral microbleed, subcortical vascular cognitive impairment

## Abstract

No study yet has compared the longitudinal course and prognosis between subcortical vascular cognitive impairment patients with and without genetic component. In this study, we compared the longitudinal changes in cerebral small vessel disease markers and cognitive function between subcortical vascular mild cognitive impairment (svMCI) patients with and without *NOTCH3* variant [*NOTCH3*(+) svMCI vs. *NOTCH3*(–) svMCI]. We prospectively recruited patients with svMCI and screened for *NOTCH3* variants by sequence analysis for mutational hotspots in the *NOTCH3* gene. Patients were annually followed-up for 5 years through clinical interviews, neuropsychological tests, and brain magnetic resonance imaging. Among 63 svMCI patients, 9 (14.3%) had either known mutations or possible pathogenic variants. The linear mixed effect models showed that the *NOTCH3*(+) svMCI group had much greater increases in the lacune and cerebral microbleed counts than the *NOTCH3*(–) svMCI group. However, there were no significant differences between the two groups regarding dementia conversion rate and neuropsychological score changes over 5 years.

## Introduction

Cerebral autosomal dominant arteriopathy with subcortical infarcts and leukoencephalopathy (CADASIL) is an autosomal dominant disorder of cerebral small vessels caused by mutations in the *NOTCH3* gene on chromosome 19 (1). CADASIL is characterized by cerebral small vessel disease (CSVD) and cognitive impairment, and is therefore considered as a genetic form of subcortical vascular cognitive impairment (SVCI). On the other hand, sporadic SVCI is mostly caused by vascular risk factors. Advanced age, hypertension (HTN), diabetes mellitus (DM), and other vascular risk factors can lead to CSVD characterized by white matter hyperintensities (WMHs), lacunes, and cerebral microbleeds (CMBs) on magnetic resonance imaging (MRI) (2).

Typically, patients with CADASIL are young at onset and have no vascular risk factors (3). However, we previously reported that approximately 13% of consecutive patients with SVCI had *NOTCH3* variants, although they were of advanced age and frequently had a history of HTN (4). In our previous cross-sectional studies, these atypical SVCI patients with *NOTCH3* variant have shown no significant differences in clinical and imaging features compared to patients without *NOTCH3* variant (4, 5). However, cross-sectional comparison, a snapshot of a single moment in time, has interpretative limitations.

To our best knowledge, no study yet has compared the longitudinal course and prognosis between SVCI patients with and without genetic component. Therefore, in this study, we compared the longitudinal changes in CSVD markers and cognitive function in subcortical vascular mild cognitive impairment (svMCI) patients with [*NOTCH3*(+) svMCI] and without [*NOTCH3*(–) svMCI] *NOTCH3* variant for a better understanding of the impact of *NOTCH3* variant.

## Materials and Methods

### Participants

We prospectively recruited 72 patients with svMCI between September 2008 and September 2011 at Samsung Medical Center in Seoul, Korea. All recruited patients met the modified Petersen's criteria for MCI as previously described (6) and had evidence of significant ischemia on their MRI scans, seen as a cap or band ≥ 10 mm and a deep white matter lesion ≥ 25 mm (modified from Fazekas ischemia criteria) (7, 8). Of the 72 svMCI patients, nine were excluded because they or their caregivers chose not to participate in the study. After a complete description of the study, written informed consent was obtained from each patient (or legally authorized representatives). The Institutional Review Board of Samsung Medical Center approved the study protocol. This study was planned and conducted in accordance with the Declaration of Helsinki. This study has been registered in the Korean Clinical Trial Registry (registration number: KCT0005516).

### Molecular Genetic Analysis

Genetic analysis was performed according to the protocols previously described (4). Peripheral blood specimens were collected after obtaining informed consent. Genomic DNA was extracted using the Wizard Genomic DNA purification kit according to the manufacturer's instructions. Mutational hotspots of the *NOTCH3* gene including exons 2–6, 8, 11, 18, 19, and 22 were sequenced. All tested exons and their exon-intron boundaries in the *NOTCH3* gene were amplified by polymerase chain reaction, as described previously (9). Cycle sequencing was performed using a BigDye Terminator Cycle Sequencing Ready Reaction kit on an ABI 3130xl Genetic Analyzer (Applied Biosystems). The nucleotides of *NOTCH3* complementary DNA were numbered according to a reference sequence (GenBank accession number: NM_000435.2). Sorting Intolerant From Tolerant (10) and Polymorphism Phenotyping (PolyPhen-2 v2.1) (11) servers were used to predict the effect of non-synonymous variants of unknown significance (VUS) on protein structure, function, phenotype, sequence conservation, and/or protein structure. In addition, 358 age- and sex-matched healthy Korean controls were screened for novel VUS in the *NOTCH3* gene using matrix-assisted laser desorption/ionization time-of-flight mass spectrometry, as described previously (12). Written informed consent was obtained from all participants including healthy controls.

Among 63 svMCI patients, nine (14.3%) had either known mutations or variants of unknown significance (VUS). Three known mutations were found in six patients: p.R544C (*n* = 4), p.R587C (*n* = 1), and p.V237M (*n* = 1). In addition, four VUS were identified in three patients: three missense variants (p.P572L, p.S947I, and p.R1175W) and one frameshift variant (p.Glu990Argfs^*^282). Two VUS (p.P572L and p.R1175W) were found in one patient. A control study of 716 chromosomes identified one variant (p.R1175W) (Supplementary Table 1). This variant might be a polymorphism rather than a pathogenic variant. These results were included in our previous report (4).

### MRI Techniques

T1, T2, 3-dimensional fluid-attenuated inversion recovery (FLAIR), and T2 Fast Field Echo-MR images were acquired using the same 3.0T MRI scanner (Philips 3.0T Achieva).

### Assessment of Lacunes and CMBs on MRI

Lacunes were defined as small lesions (≤15 and ≥3 mm in diameter) with low signal on T1-weighted images, high signal on T2-weighted images, and a perilesional halo on 80 axial slices from FLAIR images.

CMBs were defined as lesions ≤ 10 mm in diameter by using the criteria proposed by Greenberg et al. (13) on 20 axial slices of T2^*^gradient echo-MR images. CMBs were counted in four lobar regions (frontal, temporal, parietal, and occipital) and deep brain regions. The lobar regions were defined as regions ≤ 10 mm from the brain surface.

### Neuropsychological Testing

All patients underwent neuropsychological testing using the Seoul Neuropsychological Screening Battery (14, 15).

### Pittsburgh Compound B-positron Emission Tomography ([^11^C]-PiB)-PET

All patients underwent a standardized [^11^C]-PiB-PET scan at the Samsung or Asan Medical Center on a Discovery STE PET/CT scanner (GE Medical Systems, Milwaukee, WI, USA) to minimize any variance due to scanner differences. The detailed radiochemistry profiles, scanning protocol, and data analysis method were described in a previous study (8). Briefly, we calculated the PiB-uptake ratio of each voxel using the cerebellum as a reference region in the analysis. The global cortical PiB-uptake ratio was determined by combining the bilateral frontal, parietal, and temporal cortices, and the posterior cingulate gyrus. Patients were considered PiB-positive if their global PiB uptake ratio was more than 2 standard deviations (PiB retention ratio ≥ 1.5) from the mean of the normal controls.

### Follow-Up Evaluations

All patients underwent clinical interviews, a neurological examination, neuropsychological tests, brain MRI, and PiB-PET imaging at baseline. Patients were annually evaluated for 5 years through clinical interviews, neuropsychological tests, and brain MRI.

Completeness of follow-up was 63/63 (100%) at 1 year, 60/63 (95.2%) at 2 years, 55/63 (87.3%) at 3 years, 51/63 (81.0%) at 4 years, and 43/63 (68.3%) at 5 years; 7/9 (77.8%) among *NOTCH3*(+) svMCI and 36/54 (66.7%) among *NOTCH3*(–) svMCI (Supplementary Figure 1). The comparison between patients with and without complete 5-year follow-up is shown in Supplementary Table 2. There were no significant differences between the two groups except for the female ratio. The female ratio was higher in patients with complete 5-year follow-up than in those without complete follow-up.

### Statistical Analysis

To analyze the baseline differences between svMCI patients with and without *NOTCH3* variant, we used the Chi-square test or Fisher's exact test for categorical variables, and the Mann–Whitney U-test or Student *t*-test for continuous variables.

We used the linear mixed-effects model to estimate changes in CSVD markers and cognitive measures over the follow-up period. In the linear mixed-effects model, the interactions between the presence of *NOTCH3* variant and time interval (presence of *NOTCH3* variant × time) were explored to determine the influence of the presence of *NOTCH3* variant on the rate of change in CSVD markers. We controlled for age, HTN, baseline number of lacunes or CMBs, and time interval from baseline tests. To determine the trend of changes in each group, linear mixed-model analyses were separately performed in each group using age, HTN, and baseline number of lacunes or CMBs as covariates, and time interval from baseline evaluation as a predictor.

Longitudinal changes of cognitive scores in two svMCI groups were compared with linear mixed-effect models using the presence of *NOTCH3* variant and time interval (presence of *NOTCH3* variant × time) as a predictor; age, sex, education, and time interval from baseline tests were used as covariates. Because multiple cognitive scores were used for comparison, correction for multiple comparisons was performed by false discovery rate correction.

Cox regression models were used to compare the risks of progression to dementia between *NOTCH3*(+) and *NOTCH3*(–) svMCI groups after controlling for age, sex, education, and PiB positivity. Patients who did not progress to dementia were treated as censored observations from the time of their final follow-up visit.

## Results

### Baseline Characteristics and Longitudinal Follow-Up

Baseline characteristics of the subjects are shown in Table 1. There were no significant differences between the two groups with respect to age, sex ratio, education years, and prevalence of vascular risk factors. No significant group differences were seen in the baseline number of lacunes and CMBs, PiB SUVR, and mini-mental state exam (MMSE) and clinical dementia rating scale sum of boxes (CDR-SOB) scores.

**Table 1:** Baseline characteristics of patients with subcortical vascular mild cognitive impairment (svMCI), with and without *NOTCH3* variant.

	***NOTCH3*(+) svMCI**	***NOTCH3*(–) svMCI**	***p*-value**
**Number**	9	54	
**Demographics**			
Baseline age, mean ± SD (years)	70.3 ± 10.5	72.8 ± 6.3	0.519
Sex, female, *N* (%)	4 (44.4)	33 (61.1)	0.469
Education, mean ± SD (years)	8.4 ± 6.0	9.1 ± 6.3	0.610
**Vascular risk factor**, ***N*** **(%)**			
Hypertension	8 (88.9)	41 (75.9)	0.670
Diabetes mellitus	1 (11.1)	14 (25.9)	0.673
Hyperlipidemia	4 (44.4)	15 (27.8)	0.434
APOE4 carrier, *N* (%)	1 (11.1)	12 (22.2)	0.671
**Imaging markers**			
Number of lacunes, median (IQR)	8 (3–12)	3 (1–7)	0.095
Number of CMBs, median (IQR)	4 (1–16)	0 (0–5)	0.135
PiB positive (SUVR ≥ 1.5), *N* (%)	1 (11.1)	18 (33.3)	0.253
PiB SUVR, median (IQR)	1.25 (1.18–1.40)	1.35 (1.25–1.59)	0.147
**General cognition**			
MMSE, mean ± SD	25.8 ± 5.3	26.0 ± 3.0	0.488
CDR–SOB, median (IQR)	1 (0.5–1.5)	1 (0.5–1.5)	0.739
**Geriatric depression scale**, mean ± SD	10.3 ± 5.6	12.9 ± 5.8	0.222
**Follow–up duration**, mean ± SD	55.7 ± 11.6	52.5 ± 15.9	0.844
**Dementia conversion**, N (%)	1 (11.1)	12 (22.2)	0.671

*SD, Standard deviation; IQR, Interquartile range; CMB, Cerebral microbleed; SUVR, Standardized uptake value ratio; MMSE, Mini-Mental State Examination; CDR-SOB, Clinical Dementia Rating Scale Sum of Boxes*.

The mean (standard deviation) duration of follow-up were 55.7 (11.6) months in nine *NOTCH3*(+) svMCI patients and 52.5 (15.9) months in 54 *NOTCH3*(–) svMCI patients. Thirteen of 63 patients (20.6%) showed conversion to dementia on follow-up: 1/9 (11.1%) among the *NOTCH3*(+) svMCI group and 12/54 (22.2%) among the *NOTCH3*(–) svMCI group. The time to dementia diagnosis was 11 months in one *NOTCH3*(+) svMCI patient. The mean (range) time to dementia diagnosis was 31.1 (11–54) months in 12 *NOTCH3*(–) svMCI patients.

### The Impact of *NOTCH3* Variant on Longitudinal Changes of CSVD Markers

**Figure 1:**
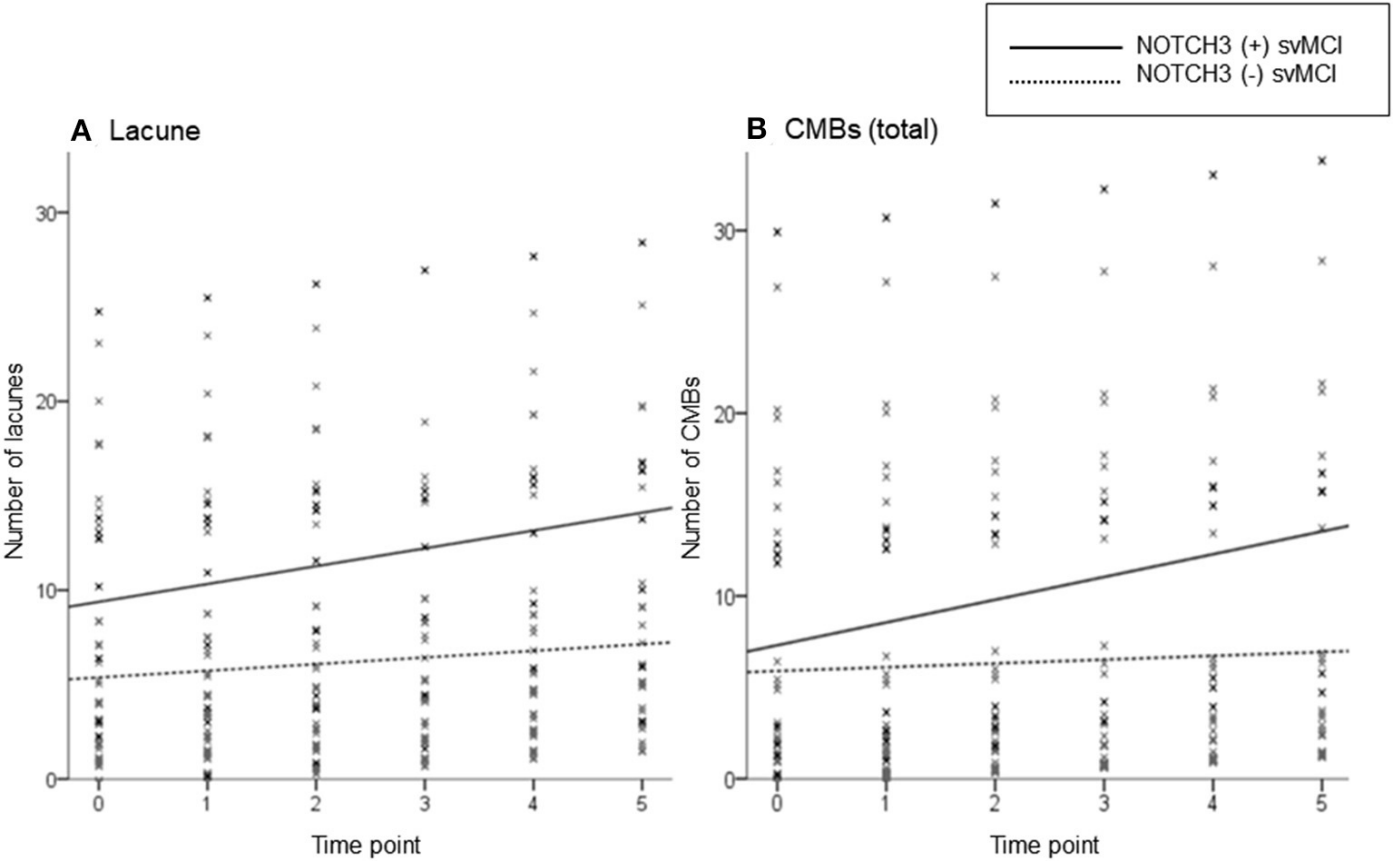
Scatterplot of the predicted number of lacunes **(A)** and cerebral microbleeds (CMBs) **(B)** in subcortical vascular mild cognitive impairment (svMCI) patients with and without *NOTCH3* variant. The solid and dotted lines show the linear regression model of patients with and without *NOTCH3* variant, respectively. Analysis controlled for age, HTN, baseline number of lacunes or CMBs, and time interval from baseline test.

Linear mixed-effect model analysis separately performed in each svMCI group showed that there were increases in lacune and CMB counts in both svMCI groups according to the time interval from baseline evaluation (Table 2). The linear mixed-effect models that tested the interactive effect of *NOTCH3* variant and time showed that the *NOTCH3*(+) svMCI group had much greater increases in lacune and CMB (total and deep) counts than *NOTCH3*(–) svMCI group after controlling for age, HTN, and baseline number of lacunes or CMBs (Table 2). Figure 1 shows the estimated effect of *NOTCH3* variant on the longitudinal changes of the number of lacunes and CMBs over a 5-year follow-up period.

**Table 2:** Comparison of longitudinal changes in the number of lacunes and cerebral microbleeds in patients with subcortical vascular mild cognitive impairment (svMCI), with and without *NOTCH3* variant.

	***NOTCH3***(+) svMCI^*^		***NOTCH3***(–) svMCI^*^		***NOTCH3*****(+) svMCI** ** vs**. ***NOTCH3*****(–) svMCI (reference)^†^**	
	***ß (SE)***	***p***	***ß (SE)***	***p***	***ß (SE)***	***p***
**Lacunes (Total)**	0.83 (0.21)	<0.001	0.37 (0.06)	<0.001	0.32 (0.15)	0.034
**CMBs**						
Total	1.24 (0.30)	<0.001	0.28 (0.09)	0.002	0.48 (0.22)	0.037
Deep	0.70 (0.24)	0.008	0.19 (0.07)	0.009	0.49 (0.18)	0.007
Lobar	0.11 (0.12)	0.336	0.13 (0.06)	0.031	0.01 (0.15)	0.961

*SE, Standard error; CMBs, Cerebral microbleeds*.

* *Results of linear mixed models separately performed in NOTCH3(+) or NOTCH3(–) svMCI group using age, HTN, and baseline number of lacunes (or CMBs) as covariates, and time interval from baseline evaluation as a predictor*.

† *Results of linear mixed models using age, HTN, baseline number of lacunes (or CMBs), and time interval from baseline tests as covariates, and the interaction between the presence of NOTCH3 mutation and time interval as a predictor*.

### The Impact of *NOTCH3* Variant on Longitudinal Cognitive Changes

In the linear mixed-effect model that tested the interactive effect of *NOTCH3* variant and time on changes of neuropsychological test scores, no neuropsychological tests showed significant differences in longitudinal change according to the presence of *NOTCH3* variant (Table 3).

**Table 3:** Comparison of longitudinal changes in neuropsychological test scores between subcortical vascular mild cognitive impairment (svMCI) patients with and without *NOTCH3* variant.

**Neuropsychological test**	***NOTCH3*****(+) svMCI** ** vs**. ***NOTCH3***(–) svMCI (reference)^*^	
	***ß (SE)***	***p***
Digit span forward	−0.06 (0.09)	1.000
Digit span backward	−0.05 (0.07)	0.916
Calculation	0.05 (0.16)	0.908
K-BNT	0.40 (0.55)	0.842
RCFT copy	0.27 (0.41)	0.773
SVLT immediate recall	0.38 (0.32)	0.711
SVLT delayed recall	0.10 (0.16)	0.681
SVLT recognition	0.40 (0.20)	0.387
RCFT immediate recall	−0.06 (0.41)	0.943
RCFT delayed recall	−0.25 (0.36)	0.792
RCFT recognition	−0.36 (0.16)	0.576
COWAT animal	0.03 (0.29)	0.909
COWAT supermarket	0.30 (0.32)	0.928
COWAT phonemic	−0.63 (0.46)	0.783
Stroop color reading	0.96 (1.49)	0.720
MMSE	0.41 (0.30)	0.634
CDR sum of boxes	−0.27 (0.18)	0.750
Geriatric depression scale	−0.34 (0.54)	0.637

*SE, standard error; K-BNT, Korean version of Boston Naming Test; RCFT, Rey–Osterrieth Figure Test; SVLT, Seoul Verbal Learning Test; COWAT, Controlled Oral Word Association Test; MMSE, Mini-mental Status Examination; CDR, Clinical Dementia Rating*.

* *Results of linear mixed models using age, sex, education, and time interval from baseline tests as covariates, and the interaction between the presence of NOTCH3 mutation and time interval as a predictor. P-values are corrected for multiple comparisons using false discovery rate correction*.

**Figure 2:**
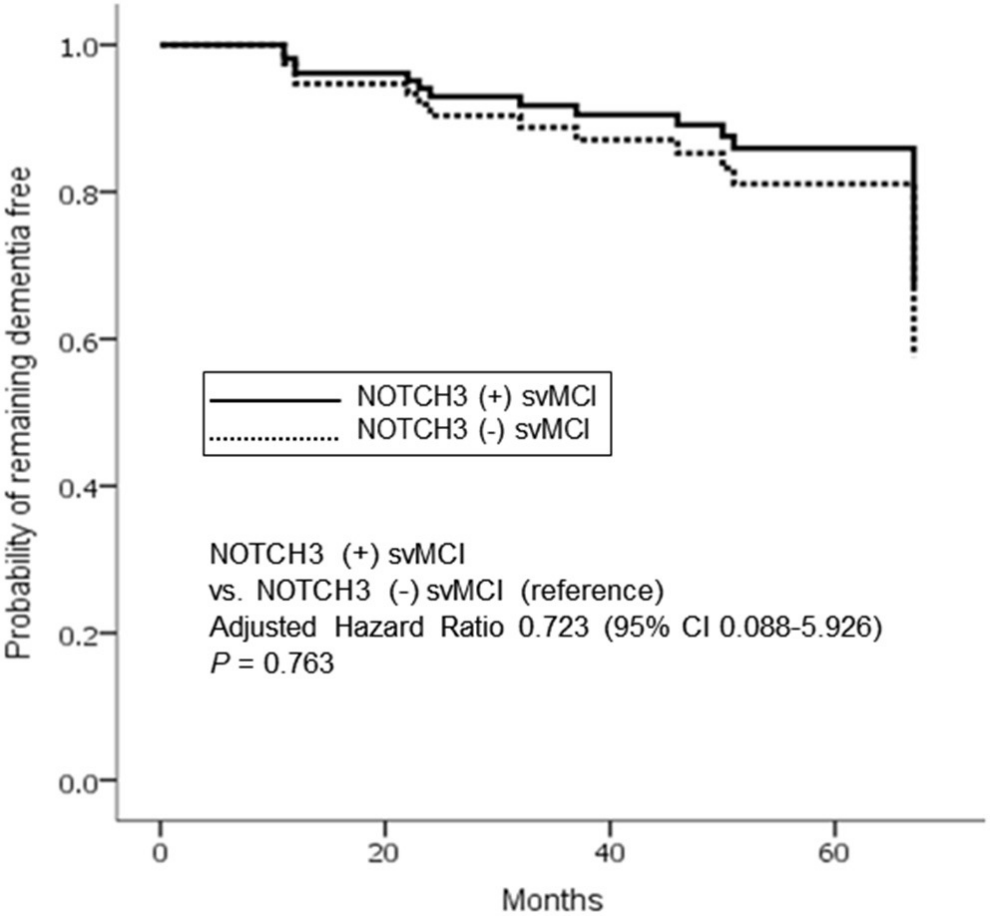
Cox proportional hazards model for progression to dementia in subcortical vascular mild cognitive impairment (svMCI) according to the presence of *NOTCH3* variant. Analysis controlled for age, sex, education, and PiB positivity. The solid and dotted lines indicate patients with and without *NOTCH3* mutation, respectively.

Cox regression model showed that dementia risk was not significantly different between *NOTCH3*(+) and *NOTCH3*(–) svMCI patients after controlling for age, sex, education, and PiB positivity (*p* = 0.763; adjusted hazard ratio, 0.723; 95% confidence interval, 0.088–5.926) (Figure 2).

## Discussion

In this prospective study, we compared the longitudinal course of svMCI patients according to the presence of *NOTCH3* variant in a well-defined svMCI cohort based on standardized imaging protocols, detailed clinical evaluation, and genetic analysis. Regarding the longitudinal changes of CSVD markers, *NOTCH3*(+) svMCI patients showed much greater increase in lacune and CMB counts than the *NOTCH3*(–) svMCI patients. However, there were no significant differences between the two groups regarding longitudinal cognitive changes including dementia conversion rate and neuropsychological score changes over 5 years. We believe this is the first study to compare the longitudinal changes of CSVD markers and cognition between SVCI patients with and without *NOTCH3* variant.

Our major finding was that the rate of increase in lacune and CMB counts was much greater in patients with *NOTCH3* variant than in those without *NOTCH3* variant. Because previous longitudinal studies have suggested that the baseline burden of CSVD is associated with the rate of change in CSVD markers (16, 17), we adjusted for baseline lacune and CMB counts. We also controlled for age and HTN as well-known risk factors for progression of CSVD. The presence of *NOTCH3* variant still remained an independent predictor for change in lacune and CMB counts.

Mutations in the *NOTCH3* gene cause degeneration of smooth muscle cells in the tunica media and thickening of the walls of cerebral small vessels (18, 19). Lacunes in CADASIL are caused by reduced microvascular perfusion due to small arteriopathy affected by *NOTCH3* mutation. Considering the similar burden of conventional vascular risk factors and additional damage to cerebral small vessels by *NOTCH3* mutation, it is understandable that *NOTCH3*(+) svMCI patients in our study showed a markedly greater increase in lacune count than *NOTCH3*(–) svMCI patients.

Both total and deep CMBs tended to increase much faster in *NOTCH3*(+) than *NOTCH3*(–) patients; however, this association was not found in lobar CMBs. In previous CADASIL studies, the distribution of CMBs is predominantly in deep location including the bilateral thalami and basal ganglia and less commonly at the gray–white junction (20–22). *NOTCH3* mutations cause arteriopathy affecting the small cerebral (deep) and leptomeningeal (superficial) penetrating arteries (23), and CMBs might result from vascular leakage of these fragile small vessels (24). Although superficial small perforating arteries associated with lobar CMBs might be also affected by *NOTCH3* mutation, the majority are thought to be deep small perforating arteries responsible for deep CMBs.

Lacunes and CMBs have been associated with cognitive impairment and decline in many previous cross-sectional (25–28) and longitudinal studies (29–32). A previous 7-year follow-up study investigating longitudinal associations between radiologic changes and cognitive decline in CADASIL patients have also shown that cognitive decline might be associated with increases in lacune and CMB counts (33). However, unexpectedly, despite a much greater increase in lacune and CMB counts in *NOTCH3*(+) svMCI patients than *NOTCH3*(–) svMCI patients, there were no significant differences in dementia conversion rate or neuropsychological score changes over 5 years between the two groups. This is likely because of similar baseline CSVD burden and inadequate differences in radiological changes to make a significant difference to cognitive decline between the two groups. Baseline CSVD burden including the number of lacunes or CMBs has been a significant predictor of cognitive decline in previous longitudinal studies (29–32). In our study, both svMCI groups with and without *NOTCH*3 variant had relatively severe CSVD burden at baseline with a similar degree between the two groups. It is also possible that a much longer follow-up is required to demonstrate significant differences in cognitive decline between the two groups. Another possible explanation is because of amyloid burden. In our study, *NOTCH3*(–) svMCI group tended to have more amyloid burden than *NOTCH3*(+) svMCI group, although it was not statistically significant. In previous studies from our group, higher amyloid uptake in svMCI was associated with more severe cognitive impairment and faster cognitive decline (34, 35). A relatively small number of *NOTCH3*(+) patients could have also attributed to these negative findings.

The strengths of our study are its prospective setting, standardized imaging protocols, and detailed clinical evaluation during follow-up. However, our results should be interpreted with caution because our *NOTCH3*(+) patients were not representative of the typical CADASIL patients (4). This was a single-center study examining a small cohort of patients and the sample size of the *NOTCH3*(+) svMCI group was particularly small. The rate of follow-up loss at 5 years was relatively high. Finally, the possibility of polymorphisms rather than pathogenic variants remains in three VUS, although these VUS were not found in 716 control chromosomes. Thus, we performed additional analyses excluding three patients with VUS and obtained similar results (Supplementary Tables 3, 4).

## Conclusion

*NOTCH3*(+) svMCI group had much greater increases in the lacune and cerebral microbleed counts than the *NOTCH3*(–) svMCI group. However, there were no significant differences between the two groups regarding dementia conversion rate and neuropsychological score changes over 5 years.

## Data Availability Statement

The datasets generated for this study will be made available on request to the corresponding author.

## Ethics Statement

The studies involving human participants were reviewed and approved by The Institutional Review Board of Samsung Medical Center. The patients/participants provided their written informed consent to participate in this study.

## Author Contributions

CY: conceptualization of the study, methodology, formal analysis, and writing - original draft. Y-EK and C-SK: methodology, validation, and formal analysis. HK: methodology and data curation. HL: methodology and formal analysis. J-HR: methodology, writing - review and editing. DN: conceptualization of the study, methodology, investigation, and supervision. SS: conceptualization of the study, methodology, formal analysis, writing-review and editing, supervision, and funding acquisition. All authors contributed to the article and approved the submitted version.

## Conflict of Interest

The authors declare that the research was conducted in the absence of any commercial or financial relationships that could be construed as a potential conflict of interest.
