# Supplementary material for: Comparison of Longitudinal Changes of Cerebral Small Vessel Disease Markers and Cognitive Function Between Subcortical Vascular Mild Cognitive Impairment With and Without NOTCH3 Variant: A 5-Year Follow-Up Study
Source: Front Neurol. 2021 Feb 25;12:586366. doi: 10.3389/fneur.2021.586366 (PMC7947323; doi:10.3389/fneur.2021.586366)
Supplement: Supplementary file 1 [file Data_Sheet_1.docx]

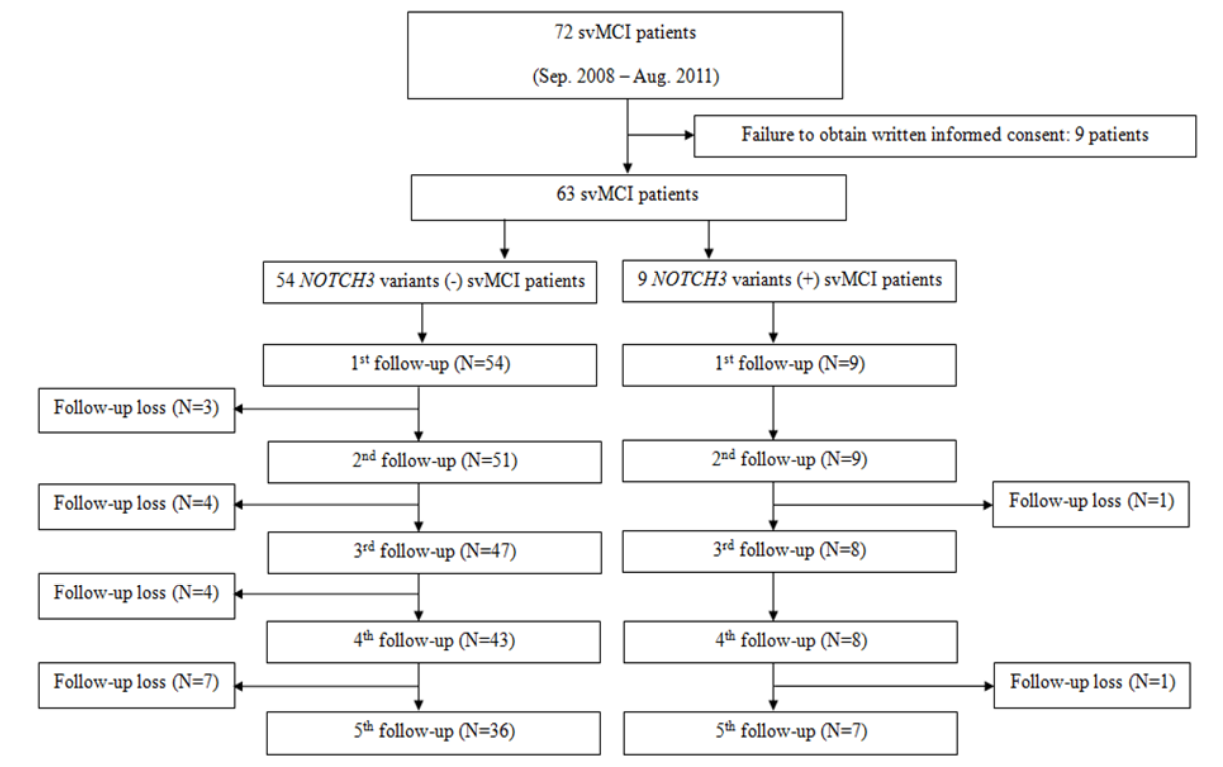


**Supplementary Figure 1. Flow chart of study participant follow-up**

**Supplementary Table 1. Variants of unknown significance (VUS) of the *NOTCH3* gene identified in three subcortical vascular mild cognitive impairment (svMCI) patients**

| Exon | Nucleotide change | Amino acid  change | No of index cases | No. of alleles  in control  chromosomes (%) | In silico analysis | | rs number |
| --- | --- | --- | --- | --- | --- | --- | --- |
|  |  |  |  |  | PolyPhen-2  (probabilistic score) | SIFT  (tolerance index) |  |
| 11 | c.1715C>T^†^ | p.P572L | 1 | 0/716 (0) | Probably damaging (0.99) | Tolerable (0.12) | NA |
| 18 | c.2968delG | p.E990Rfs*282 | 1 | 0/716 (0) | NA | NA | NA |
|  | c.2840G>T | p.S947I | 1 | 0/716 (0) | Probably damaging (1.00) | Tolerable (0.11) | NA |
| 22 | c.3523C>T^†,‡^ | p.R1175W | 1 | 8/716 (1.12) | Probably damaging (0.994) | Not tolerable (0.02) | rs200504060 |

^†^These two VUS were identified in one patient.

^‡^A variant that was identified in a control study was considered a polymorphism rather than a pathogenic mutation

**Supplementary Table 2. Comparison between patients with and without complete 5-year follow –up**

|  | Complete 5-year follow -up | Loss to 5-year follow-up | *p* value |
| --- | --- | --- | --- |
| *Number* | 43 | 20 |  |
| *NOTCH3*(+)*, N (%)* | 7 (16.3) | 2 (10.0) | 0.706 |
| *Demographics* |  |  |  |
| Baseline age, mean ± SD (years) | 71.6 ± 7.3 | 74.3 ± 5.8 | 0.277 |
| Sex, female, N (%) | 30 (69.8) | 7 (35.0) | 0.013 |
| Education, mean ± SD (years) | 8.2 ± 4.9 | 10.9 ± 5.8 | 0.064 |
| *Vascular risk factor, N (%)* |  |  |  |
| Hypertension | 35 (81.4) | 14 (70.0) | 0.342 |
| Diabetes mellitus | 12 (27.9) | 3 (15.0) | 0.349 |
| Hyperlipidemia | 13 (30.2) | 6 (30.0) | 1.000 |
| *APOE4 carrier, N (%)* | 6 (14.0) | 7 (35.0) | 0.092 |
| *Imaging markers* |  |  |  |
| Number of lacunes, median (IQR) | 3 (1-8) | 4 (1–10) | 0.941 |
| Number of CMBs, median (IQR) | 1 (0-5) | 1 (0–5) | 0.733 |
| PiB positive (SUVR≥1.5), N (%) | 11 (25.6) | 8 (42.1) | 0.238 |
| PiB SUVR, median (IQR) | 1.30 (1.25–1.49) | 1.39 (1.23–2.08) | 0.317 |
| *General cognition* |  |  |  |
| MMSE, mean ± SD | 25.9 ± 3.7 | 26.0 ± 2.7 | 0.644 |
| CDR–SOB, median (IQR) | 1 (0.5–1.5) | 1 (0.5–1.5) | 0.721 |
| *Geriatric Depression scale, mean ± SD* | 12.4 ± 5.7 | 12.8 ± 6.1 | 0.813 |

SD, Standard deviation; IQR, Interquartile range; CMB, Cerebral microbleed; SUVR, Standardized uptake value ratio; MMSE, Mini-Mental State Examination; CDR-SOB, Clinical Dementia Rating Scale Sum of Boxes.

**Supplementary Table 3. Comparison of longitudinal changes in the number of lacunes and cerebral microbleeds in patients with subcortical vascular mild cognitive impairment (svMCI) with and without *NOTCH3* mutation (excluding 3 patients with variants of unknown significance)**

|  | *NOTCH3*(+) svMCI^*^ | | *NOTCH3*(-) svMCI^*^ | | *NOTCH3*(+) svMCI  vs. *NOTCH3*(-) svMCI (reference)^†^ | |
| --- | --- | --- | --- | --- | --- | --- |
|  | *ß (SE)* | *p* | *ß (SE)* | *p* | *ß (SE)* | *p* |
| Lacunes (Total) | 0.85 (0.29) | 0.014 | 0.37 (0.06) | < 0.001 | 0.52 (0.18) | 0.004 |
| CMBs |  |  |  |  |  |  |
| Total | 1.33 (0.44) | 0.013 | 0.28 (0.09) | 0.002 | 0.49 (0.26) | 0.066 |
| Deep | 0.85 (0.33) | 0.043 | 0.19 (0.07) | 0.009 | 0.66 (0.22) | 0.003 |
| Lobar | 0.02 (0.06) | 0.722 | 0.13 (0.06) | 0.031 | -0.15 (0.18) | 0.418 |

SE, Standard error; CMBs, Cerebral microbleeds.

*Results of linear mixed models separately performed in *NOTCH3*(+) or *NOTCH3*(−) svMCI group using age, HTN, and baseline number of lacunes (or CMBs) as covariates, and time interval from baseline evaluation as a predictor.

^†^Results of linear mixed models using age, HTN, baseline number of lacunes (or CMBs), and time interval from baseline tests as covariates, and the interaction between the presence of *NOTCH3* mutation and time interval as a predictor.

**Supplementary Table 4. Comparison of longitudinal changes in neuropsychological test scores between subcortical vascular mild cognitive impairment (svMCI) patients with and without *NOTCH3* mutation (excluding 3 patients with variants of unknown significance)**

| Neuropsychological test | *NOTCH3*(+) svMCI  vs. *NOTCH3*(-) svMCI (reference)^*^ | |
| --- | --- | --- |
|  | *ß (SE)* | *p* |
| Digit span forward | -0.06 (0.11) | 0.784 |
| Digit span backward | -0.05 (0.08) | 0.800 |
| Calculation | 0.00 (0.20) | 0.983 |
| K-BNT | 0.64 (0.70) | 0.732 |
| RCFT copy | 0.90 (0.51) | 0.492 |
| SVLT immediate recall | 0.70 (0.41) | 0.423 |
| SVLT delayed recall | 0.40 (0.20) | 0.954 |
| SVLT recognition | 0.49 (0.25) | 0.486 |
| RCFT immediate recall | 0.40 (0.52) | 0.725 |
| RCFT delayed recall | 0.23 (0.44) | 0.765 |
| RCFT recognition | -0.21 (0.20) | 0.810 |
| COWAT animal | 0.33 (0.37) | 0.666 |
| COWAT supermarket | 0.09 (0.41) | 0.880 |
| COWAT phonemic | -0.27 (0.55) | 0.703 |
| Stroop color reading | 0.87 (1.66) | 0.720 |
| MMSE | 0.53 (0.39) | 0.516 |
| CDR Sum of Boxes | -0.33 (0.22) | 0.482 |
| Geriatric depression scale | -0.69 (0.70) | 0.734 |

SE, standard error; K-BNT, Korean version of Boston Naming Test; RCFT, Rey–Osterrieth Figure Test; SVLT, Seoul Verbal Learning Test; COWAT, Controlled Oral Word Association Test; MMSE, Mini-mental Status Examination; CDR = Clinical Dementia Rating.

^*^Results of linear mixed models using age, sex, education, and time interval from baseline tests as covariates, and the interaction between the presence of *NOTCH3* mutation and time interval as a predictor. *P* values are corrected for multiple comparisons using false discovery rate correction.
